# Comment on “Using novel oxidative phosphorylation inhibitors to attenuate drug resistance in human gliomas” by Tsai et al. (2025)

**DOI:** 10.17179/excli2025-8694

**Published:** 2025-10-21

**Authors:** Abdullah Saad, Rameen Khan

**Affiliations:** 1 Sindh Medical College, Jinnah Sindh Medical University, Karachi 75510, Pakistan

## ⁯

Glioblastoma multiforme (GBM) represents the most aggressive form of primary brain tumors, characterized by high recurrence rates, poor prognosis, and limited therapeutic options. Standard-of-care treatments, including surgical resection, radiotherapy, and temozolomide (TMZ) chemotherapy, provide only modest survival benefits due to the rapid development of resistance mechanisms. This clinical challenge underscores the urgency for novel therapeutic strategies aimed at overcoming these resistance pathways and improving patient outcomes. In this context, Tsai et al. (2025[8]) have presented an insightful study demonstrating the potential of Gboxin, an oxidative phosphorylation (OXPHOS) inhibitor, in attenuating TMZ resistance in GBM.

The metabolic heterogeneity and adaptability of GBM contribute significantly to its therapeutic resistance (Hanahan and Weinberg, 2011[3]; Martinez-Outschoorn et al., 2017[4]; Barbieri and Kouzarides, 2020[1]). Hanahan and Weinberg (2011[3]) laid the foundation for understanding cancer hallmarks, highlighting how metabolic reprogramming supports tumor survival, treatment resistance, and proliferation. Their seminal work established the notion that metabolic changes are not mere by-products of oncogenesis but are essential for cancer progression. Expanding on this, Martinez-Outschoorn et al. (2017[4]) provided a detailed perspective on how cancer cells actively manipulate metabolic pathways to promote growth and survival under stress conditions, including exposure to chemotherapy. By selectively targeting OXPHOS, Gboxin disrupts mitochondrial energy production, which is a vital process for tumor cells, leading to impaired proliferation, enhanced apoptosis, and tumor growth suppression in both TMZ-sensitive and TMZ-resistant GBM models (Shi et al., 2019[7]; Tsai et al., 2025[8]). The study by Shi et al. (2019[7]) was instrumental in characterizing Gboxin's mechanism of action, revealing its selective toxicity towards cancer cells dependent on mitochondrial respiration, while sparing non-transformed cells. This selectivity highlights Gboxin's potential as a targeted anti-cancer agent with reduced off-target toxicity.

Furthermore, the identification of polo-like kinase 2 (PLK2) as a downstream target by Tsai et al. (2025[8]) enhances the translational relevance of their findings, given PLK2's established association with glioma progression, therapeutic resistance, and poor prognosis (Ding et al., 2022[2]). Ding et al. (2022[2]) emphasized the role of PLK2 as a key regulator of glioma cell proliferation and survival, suggesting its utility both as a therapeutic target and a prognostic biomarker. These findings not only reinforce the therapeutic potential of Gboxin but also introduce the possibility of utilizing PLK2 as a companion biomarker for patient selection and monitoring therapeutic response.

Nevertheless, GBM's metabolic plasticity enables tumor cells to activate compensatory pathways that circumvent therapeutic interventions, underscoring the need for combinatorial strategies to mitigate resistance emergence (Martinez-Outschoorn et al., 2017[4]; Ohshima and Morii, 2021[5]; Pavlova and Thompson, 2016[6]). Ohshima and Morii (2021[5]) detailed the complexity of metabolic reprogramming during tumor progression, highlighting how cancer cells dynamically adjust their metabolic phenotype to adapt to therapeutic pressures and unfavorable microenvironments. Similarly, Pavlova and Thompson (2016[6]) identified emerging hallmarks of cancer metabolism, emphasizing how metabolic flexibility and the ability to switch between glycolysis and oxidative phosphorylation underpins tumor survival and therapeutic resistance. Furthermore, heterogeneity in apoptosis induction among GBM cell lines observed by Tsai et al. (2025[8]) highlights the importance of biomarker-driven patient stratification to maximize therapeutic efficacy and minimize treatment failure.

The integration of OXPHOS inhibitors like Gboxin into multimodal treatment regimens, coupled with robust biomarker identification, represents a promising avenue to overcome therapeutic resistance in GBM. However, future investigations should elucidate the pharmacodynamics, safety profile, and long-term efficacy of Gboxin in clinically relevant models, to facilitate its potential clinical translation. Additionally, exploring metabolic vulnerabilities beyond OXPHOS, including targeting pathways such as glycolysis, glutaminolysis, and lipid metabolism, in combination with Gboxin, could further enhance therapeutic outcomes and prevent resistance relapse. It is also essential to consider the tumor microenvironment's role, including hypoxia-induced metabolic reprogramming, which can influence the efficacy of metabolic inhibitors like Gboxin.

In conclusion, the study by Tsai et al. (2025[8]) advances the paradigm of metabolic targeting in GBM and provides a compelling preclinical rationale for further exploration of Gboxin as a therapeutic adjunct. Their work contributes to the growing body of evidence supporting metabolic intervention as a viable strategy to overcome therapeutic resistance in aggressive malignancies like GBM.

## Declaration

### Disclosure of AI use

Portions of this manuscript were enhanced using ChatGPT for improving readability. All AI-generated content was reviewed and verified by the authors.

### Conflict of interest

The authors declare no conflict of interest.
